# Peripapillary Retinal Nerve Fiber Layer Thickness and the Evolution of Cognitive Performance in an Elderly Population

**DOI:** 10.3389/fneur.2017.00093

**Published:** 2017-03-21

**Authors:** Juan Luis Méndez-Gómez, Marie-Bénédicte Rougier, Laury Tellouck, Jean-François Korobelnik, Cédric Schweitzer, Marie-Noëlle Delyfer, Hélène Amieva, Jean-François Dartigues, Cécile Delcourt, Catherine Helmer

**Affiliations:** ^1^University Bordeaux, INSERM, Bordeaux Population Health Research Center, Team LEHA, UMR 1219, Bordeaux, France; ^2^University Hospital, Ophthalmology, Bordeaux, France; ^3^University Hospital, Memory Consultation, CMRR, Bordeaux, France

**Keywords:** retinal nerve fiber layer, episodic memory, cognition, elderly, optical coherence tomography

## Abstract

Retinal nerve fiber layer (RNFL) thickness is reduced in Alzheimer’s patients. However, whether it is associated with early evolution of cognitive function is unknown. Within 427 participants from the Three-City-Alienor longitudinal population-based cohort, we explored the relationship between peripapillary RNFL thicknesses and the evolution of cognitive performance. RNFL was assessed at baseline by spectral domain optical coherence tomography; cognitive performances were assessed at baseline and at 2 years, with the Mini–Mental State Examination, the Isaacs’ set test, and the Free and Cued Selective Reminding Test (FCSRT). Multivariate linear mixed models were performed. The RNFL was not associated with initial cognitive performance. Nevertheless, a thicker RNFL was significantly associated with a better cognitive evolution over time in the *free delayed recall* (*p* = 0.0037) and *free* + *cued delayed recall* (*p* = 0.0043) scores of the FCSRT, particularly in the temporal, superotemporal, and inferotemporal segments. No associations were found with other cognitive tests. The RNFL was associated with changes in scores that assess episodic memory. RNFL thickness could reflect a higher risk of developing cognitive impairment over time.

## Introduction

Alzheimer’s disease (AD) is associated with brain neurodegeneration, particularly in the medial temporal area, which leads to cognitive decline and dementia. These lesions occur several years before the clinical phase of dementia (1, 2). Detection of this neurodegenerative process at an early stage could allow prediction of cognitive decline in subsequent years.

The retina and brain are intimately linked as a result of common embryonic origin. The eye is a sensory organ that is truly part of the central nervous system, with neuronal cells that could be susceptible to degeneration, directly or indirectly. Thus, the condition of nerve fibers in the eye could reflect the condition of nerve fibers in the brain (3). Spectral domain optical coherence tomography (SD-OCT) allows easy and accurate evaluation of the peripapillary retinal nerve fiber layer (RNFL), by measuring the thickness of this layer containing ganglion cell axons in a circle around the optic nerve. This RNFL could thus enable evaluation of neurodegeneration in mild cognitive impairment (MCI) and AD pathology (4).

Abnormality in RNFL thickness has been described in several neurodegenerative conditions, in particular multiple sclerosis, AD, and Parkinson’s disease (5–7). Regarding MCI and/or AD, several previous studies have demonstrated reduced RNFL thickness compared to that of a healthy control population, initially in histological studies (8) and then in OCT studies (4, 9, 10); these studies were, however, mostly cross-sectional case–control studies. Additionally, we previously found an association between glaucoma pathology, which has reduced RNFL thickness, and the occurrence of dementia 3 years later (11). However, except for one study with a small sample (12), the association between RNFL and the evolution of cognitive function has not yet been studied in a general elderly population.

Our study aims to explore the relationship between peripapillary RNFL thickness measured by SD-OCT and the evolution of cognitive function in several cognitive domains measured over a 2-year period in the elderly population.

## Materials and Methods

### Study Population

**Figure 1:**
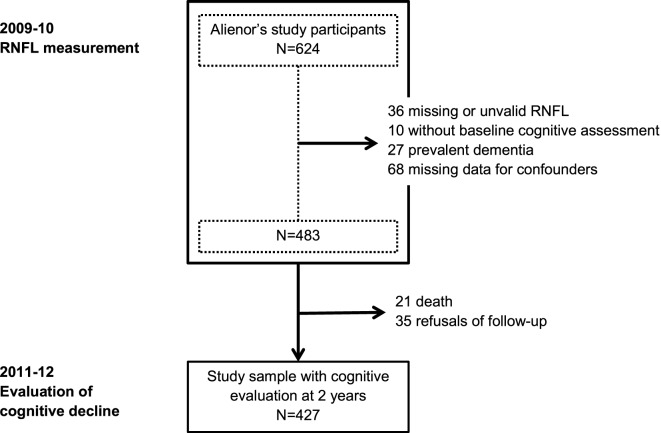
**Flow chart of the population**. RNFL, retinal nerve fiber layer.

The Three-City (3C) study is a prospective population-based cohort, which aims to estimate the risk of dementia and cognitive impairment attributable to vascular factors. At baseline (1999–2001), 9,294 community-dwelling French adults aged ≥65 years in three French cities (Bordeaux, Dijon, Montpellier) were enrolled, including 2,104 from Bordeaux. The methodology has been described elsewhere (13). Data regarding sociodemographic characteristics, lifestyle, physical and mental health, medications, disability, and cognitive functions were assessed at baseline and subsequently 2, 4, 7, 10, and 12 years later. At the 7-year follow-up (in 2006–2008), 963 participants from Bordeaux agreed to participate to the Alienor study (Antioxydants, Lipides Essentiels, Nutrition et maladies OculaiRes), consisting of an ophthalmological examination (14). These participants were then followed-up every two years with eye examinations concurrent with cognitive evaluations. In 2009–2010, a SD-OCT examination of the optic nerve was included in the eye examination; 624 participants were evaluated. Our population consists of participants with (i) valid RNFL measurements at that time, i.e., without any segmentation problem and without ocular pathology that could influence RNFL measurement (vitreomacular traction, myelinated retinal nerve fibers, peripapillary choroidal neovascularization, or myopic chorioretinopathy), (ii) concurrent cognitive evaluations, (iii) without prevalent dementia in 2009–2010, and (iv) without missing data for confounders. Because glaucoma is a degenerative disease of the optic nerve that could have common pathophysiological mechanisms with AD, participants with this disease were retained in the study population, and glaucoma was included as a covariate in the analyses (11). Finally, participants without cognitive evaluation at 2 years were excluded, with thus 427 participants considered for the present study (Figure 1).

Approvals were obtained from the Ethical Committee Sud-Ouest et Outre-Mer III for the Alienor study and from the Ethical Committee of the University Hospital of Kremlin-Bicêtre and Sud-Mediterranée III for the 3C study. All participants signed an informed consent.

### Cognitive Assessment

At each follow-up visit, trained psychologists assessed the cognitive function of each participant during face-to-face visits using the Mini–Mental State Examination (MMSE) (15), the Isaacs’ set test at 30 s (16) and the Free and Cued Selective Reminding Test (FCSRT) (17). After the neuropsychological examination, participants suspected of having dementia were visited by a neurologist or a geriatric specialist, to confirm the diagnosis. Finally, an independent committee of neurologists reviewed all potential cases of dementia with all available information in order to obtain a consensus on the diagnosis, according to the DSM-IV criteria for dementia (18). For this study, we analyzed RNFL measurement in relation to cognitive performance assessed at the same baseline time as RNFL and 2 years later.

The MMSE is a composite scale from 0 to 30 points that evaluates the global cognitive state. The Isaacs’ set test evaluates categorical verbal fluency by measuring the ability to generate word lists from four semantic categories (animals, fruits, colors, and cities) in 30 s. Finally, the FCSRT evaluates episodic memory using a list of 16 words belonging to 16 semantic categories. This test assesses episodic memory processes by controlling the encoding and retrieval conditions. It starts with an initial phase of categorical semantic learning, followed by three notation steps (*immediate recall, three free/cued recalls*, and *delayed free/cued recalls*). We used four variables in our study: the *three free recalls* score, the added score of *three free plus cued recalls*, the *delayed free recalls*, and finally the added score of *delayed free plus cued recalls*. Other details of the procedure have been previously described (19). None of the staff involved in cognitive testing had access to ophthalmologic data.

### Ophthalmologic Assessments

#### SD-OCT Measures

**Figure 2:**
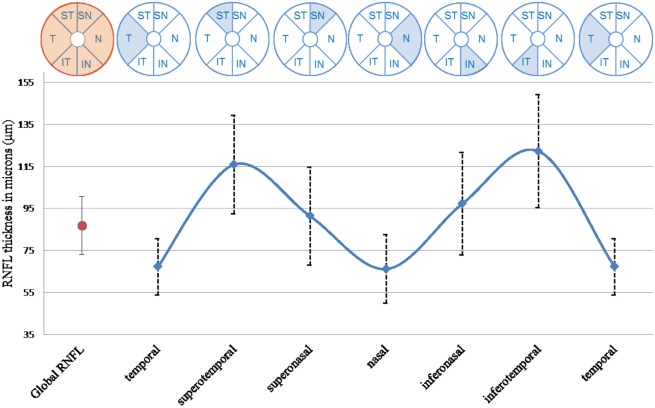
**Global and six segments thicknesses of RNFL, *n* = 427**. T, temporal segment; ST, superotemporal segment; SN, superonasal segment; N, nasal segment; IN, inferonasal segment; IT, inferotemporal segment.

The OCT examination was performed using SPECTRALIS (Software Version 5.4.7.0; Heidelberg Engineering, Heidelberg, Germany) without pupil dilation by one experienced technician. The device acquisition rate of OCT images was 40,000 A-scans/s. The OCT provides an automatic real-time (ART) function that adjusts for eye movement and increases image quality. Details of our OCT measures have been previously described (20). The peripapillary RNFL thickness was acquired using the following conditions (14): resolution mode: high speed; circle diameter: 3.5 mm; size X: 768 pixels (10.9 mm); size Z: 496 pixels (1.9 mm); scaling X: 14.14 lm/pixel; scaling Z: 3.87 lm/pixel; and ART mode: 16 images. The minimum reliable value of global RNFL thickness was retained for analysis when the measure was present in both eyes; otherwise, data from the eye with the only reliable value were retained. RNFL thicknesses in the six segments measured by the device (superotemporal, temporal, inferotemporal, inferonasal, nasal, and superonasal) were retained for the same eye (Figure 2).

#### Other Ophthalmologic Variables

As previously described (14), the diagnosis of glaucoma was established according to the classification proposed by Foster et al. (21). Cataract surgery was assessed during eye examination. Optic disc size (vertical disc diameter) was measured using optic disc photography in duplicate by two trained graders and by a glaucoma specialist if there was inconsistency between the first two interpretations. Finally, axial length was measured during a subsequent visit in a subsample of participants using non-contact partial coherence laser interferometry (IOL Master; Carl Zeiss Meditec AG, Jena, Germany). Five measurements were taken per eye, and the mean was calculated. None of the staff involved in eye examination had access to the cognitive data.

### Other Variables

The following covariates were assessed: age, sex, education level (elementary school, secondary school vs higher level), the presence of Apolipoprotein ε4 allele and diabetes (antidiabetic treatment and/or a fasting glucose level ≥7 mmol/L or a non-fasting glucose level ≥11 mmol/L).

### Data Analysis

Baseline characteristics of the study sample were first described. To study the relationship between the RNFL thickness and the cognitive scores at baseline and cognitive change over time we built linear mixed models to account for the correlations due to repeated cognitive measures (22). We first analyzed the relationship between global RNFL thickness (continuous variable) and each cognitive test to avoid multiplicity of statistical analyses. We further analyzed all six RNFL segments only when the result was significant for global RNFL. Due to ceiling and floor effects and curvilinearity (unequal interval scaling) for MMSE and FCSRT scores, we used a previously published procedure that corrects the metrological properties to normalize the score distributions (23). First, models were adjusted for age, sex, time, education level, ApoE4, diabetes, cataract surgery, optic disc size, and glaucoma in multivariable linear mixed models (Model A). The interaction between each variable and time was systematically tested and retained in the model when *p* < 0.10. Next, given the knowledge that RNFL thickness decreases with increasing axial length (24), a second model (Model B) was built including axial length as a covariate; this measure was available for only a subset (*n* = 204) of the study population. Sensitivity analyses excluding participants with glaucoma were performed. In complementary analyses, we re-ran the analyses for global RNFL considering RNFL as a binary variable, according to the classification provided by the Spectralis software: outside normal limits (lowest 1% of the distribution), borderline (bottom 1–5% of the distribution), and within normal (above 5%) based on the age-matched comparison with an internal normative database of 201 healthy eyes of Caucasian patients. Thus, we analyzed the evolution of cognitive performance for participants with borderline value or value outside normal limits, compared to value within normal.

The normalization procedure of cognitive scores was performed using R software (version 2.14.1, 2011© The R Foundation for Statistical Computing). All other statistical procedures were performed using SAS software (version 9.3, SAS Institute Inc., Cary, NC, USA).

## Results

Population characteristics of the 427 participants are described in Table 1. The mean age was 81.6 (SD: 4.2) years and 62.3% were women. The mean global RNFL thickness was 86.8 μm (SD: 13.7). As expected, the inferotemporal (122.3 μm) and superotemporal (115.9 μm) segments were thickest. Mean MMSE score at baseline was 27.8 (SD: 1.9). Cognitive performance significantly declined over the 2-year follow-up, irrespective of the test considered (Table S1 in Supplementary Material).

**Table 1:** **Characteristics of the study population at inclusion, *N* = 427**.

	Mean or *n*	SD or %
**Sociodemographic, medical, and ophthalmological characteristics**		
Age (years), mean (SD)	81.6	4.2
Female, *n* (%)	266	62.3
Educational level, *n* (%)		
Elementary school	33	7.7
Secondary school	198	46.4
Higher level	196	45.9
Apolipoprotein ε4, *n* (%)	72	16.9
Diabetes, *n* (%)	44	10.3
Glaucoma, *n* (%)	26	6.1
Cataract surgery (with or without implant), *n* (%)	212	49.6
Vertical diameter of the ONH (in mm), mean (SD)	2.0	0.2
Axial length^a^ (in mm), mean (SD)	23.4	1.2
**Cognitive assessment tests at baseline^b^**		
MMSE, mean (SD)	27.8	1.9
Isaacs’ set test at 30″, mean (SD)	45.1	9.7
FCSRT, mean (SD)		
Score of *three free recalls*	26.1	7.1
Score of *three free plus cued recalls*	45.1	4.6
Score of *delayed free recall*	10.4	3.1
Score of *delayed free plus cued recalls*	15.3	1.6
**RNFL assessment measures**		
Global RNFL thickness (in μm), mean (SD)	86.8	13.7
Global RNFL classification,^c^ *n* (%)		
Within normal limits	297	69.6
Borderline	54	12.6
Outside normal limits	76	17.8
Segments thickness (in μm), mean (SD)
Superotemporal	115.9	23.5
Temporal	67.2	13.4
Inferotemporal	122.3	27.1
Inferonasal	97.3	24.4
Nasal	66.2	16.4
Superonasal	91.4	23.3

*MMSE, Mini–Mental State Examination; FCSRT, Free and Cued Selective Reminding Test; RNFL, retinal nerve fiber layer*.

*Missing data: Isaacs’ set test at 30″ (*n* = 5); all FCSRT scores (*n* = 30)*.

*^a^Measure obtained from 204 participants*.

*^b^Scores for the cognitive assessment performed at baseline, at the same time as RNFL measurement, are presented here*.

*^c^Classification provided by the Spectralis software: outside normal limits (lowest 1% of the distribution), borderline (bottom 1–5% of the distribution) and within normal (above 5%) based on the age-matched comparison with an internal normative database of 201 healthy eyes of Caucasian patients. For each participant, the worse eye is considered here*.

The global RNFL was not associated with initial cognitive performance regardless of cognitive test considered (Table 2). Global RNFL was not associated with cognitive evolution based on the MMSE or the Isaacs’ set test at follow-up (RNFL × time term). However, global RNFL was associated with the two FCSRT scores of delayed recall. Specifically, a thicker global RNFL was associated with a lower decline of *delayed free recall* (β = 0.015; 95% CI, 0.005 to 0.025; *p* = 0.004 for 1 μm RNFL thickness) and *delayed free plus cued recalls* (β = 0.007; 95% CI, 0.002 to 0.011; *p* = 0.004) (Table 2, model A).

**Table 2:** **Initial differences and evolution over time of the neuropsychological tests according to the global thickness of the RNFL**.

	Model A (*n* = 427)		Model B (*n* = 204)	
	β (95% CI)	*p*	β (95% CI)	*p*
**MMSE**				
RNFL	−0.006 (−0.020 to 0.007)	0.358	0.008 (−0.012 to 0.027)	0.434
RNFL × time	0.004 (−0.003 to 0.011)	0.234	0.002 (−0.007 to 0.011)	0.692
**Isaacs’ set test at 30″**				
RNFL	−0.001 (−0.069 to 0.067)	0.986	0.004 (−0.090 to 0.098)	0.933
RNFL × time	0.001 (−0.020 to 0.023)	0.910	0.017 (−0.013 to 0.046)	0.264
**FCSRT**				
Score of *three free recalls*^a^				
RNFL	0.011 (−0.004 to 0.025)	0.153	0.015 (−0.005 to 0.036)	0.138
RNFL × time	0.001 (−0.004 to 0.007)	0.609	0.004 (−0.004 to 0.011)	0.342
Score of *three free plus cued recalls*^b^				
RNFL	0.010 (−0.001 to 0.021)	0.089	0.013 (−0.003 to 0.029)	0.109
RNFL × time	−0.001 (−0.006 to 0.004)	0.662	−0.002 (−0.008 to 0.005)	0.589
Score of *delayed free recall*^c^				
RNFL	−0.002 (−0.028 to 0.024)	0.862	−0.003 (−0.040 to 0.034)	0.869
RNFL × time	0.015 (0.005 to 0.025)	**0.004**	0.017 (0.003 to 0.030)	**0.014**
Score of *delayed free plus cued recalls*				
RNFL	−0.006 (−0.017 to 0.004)	0.253	−0.010 (−0.024 to 0.005)	0.195
RNFL × time	0.007 (0.002 to 0.011)	**0.004**	0.011 (0.005 to 0.017)	**<0.001**

*Model A adjusted for: time, age, interaction age/time, sex, school level, Apolipoprotein ε4, diabetes, diameter of optic nerve head, cataract surgery, and glaucoma*.

*Model B adjusted for: the same A-model with additional adjustment for axial length (AL) measure*.

*^a^Additional adjustment for the interaction sex/time in model A and B*.

*^b^Additional adjustment for the interaction diabetes/time in model A and B and the interaction AL/time in model B*.

*^c^Additional adjustment for the interaction Apolipoprotein ε4/time in model A and B*.

*MMSE, Mini–Mental State Examination; FCSRT, Free and Cued Selective Reminding Test; RNFL, retinal nerve fiber layer*.

*Bold p-value indicates p < 0.05*.

These associations remained unchanged after additional adjustment for axial length in the subset which had this measure (Table 2, model B). Mean delayed FCSRT scores, at baseline and at the follow-up, are graphically represented in Figure S1 in Supplementary Material according to baseline RNFL value.

For FCSRT scores showing significant association with global RNFL, the relationships with each of the six RNFL segments were analyzed (Table 3). The temporal segments had the strongest association with the two FCSRT delayed recall scores. Thicker RNFL of the temporal, inferotemporal, and superotemporal segments was significantly associated with a lower decline in the *delayed free recall* score during follow-up (β = 0.013; 95% CI, 0.003 to 0.023; *p* = 0.011; β = 0.007; 95% CI, 0.002 to 0.012; *p* = 0.005; and β = 0.007; 95% CI, 0.002 to 0.013; *p* = 0.013, respectively) in model A. The same trends were observed in model B after additional adjustment for axial length; although, the associations were only borderline significant for the temporal and inferotemporal segments. Surprisingly, the temporal segment also showed an association with a lower initial score (β = −0.029; 95% CI, −0.054 to −0.005; *p* = 0.020), but this association was no longer significant after adjustment for axial length.

**Table 3:** **Initial differences and evolution over time of the FCSRT score of delayed free recall and delayed free plus cued recalls according to the segments of the RNFL**.

	Model A (*n* = 427)		Model B (*n* = 204)	
	β (95% CI)	*p*	β (95% CI)	*p*
**Delayed free recall^a^**				
Superotemporal	−0.009 (−0.024 to 0.006)	0.223	−0.009 (−0.031 to 0.012)	0.402
Superotemporal × time	0.007 (0.002 to 0.013)	**0.013**	0.012 (0.004 to 0.020)	**0.003**
Temporal	−0.029 (−0.054 to −0.005)	**0.020**	−0.024 (−0.060 to 0.011)	0.183
Temporal × time	0.013 (0.003 to 0.023)	**0.011**	0.013 (−0.002 to 0.027)	0.080
Inferotemporal	−0.006 (−0.020 to 0.007)	0.343	−0.009 (−0.028 to 0.010)	0.340
Inferotemporal × time	0.007 (0.002 to 0.012)	**0.005**	0.006 (−0.0004 to 0.013)	0.067
Inferonasal	0.009 (−0.005 to 0.023)	0.192	0.005 (−0.014 to 0.024)	0.578
Inferonasal × time	0.005 (−0.001 to 0.011)	0.084	0.006 (−0.002 to 0.013)	0.142
Nasal	0.020 (−0.00002 to 0.040)	0.050	0.023 (−0.005 to 0.052)	0.112
Nasal × time	0.005 (−0.003 to 0.013)	0.232	0.005 (−0.006 to 0.017)	0.351
Superonasal	−0.003 (−0.018 to 0.011)	0.648	−0.008 (−0.028 to 0.013)	0.457
Superonasal × time	0.004 (−0.002 to 0.010)	0.169	0.006 (−0.002 to 0.014)	0.143
**Delayed free plus cued recalls**				
Superotemporal	−0.005 (−0.011 to 0.001)	0.112	−0.005 (−0.014 to 0.003)	0.232
Superotemporal × time	0.002 (−0.001 to 0.005)	0.108	0.005 (0.001 to 0.009)	**0.010**
Temporal	−0.009 (−0.019 to 0.001)	0.089	−0.012 (−0.026 to 0.002)	0.096
Temporal × time	0.005 (−0.00003 to 0.009)	0.051	0.009 (0.002 to 0.016)	**0.010**
Inferotemporal	−0.005 (−0.010 to 0.001)	0.092	−0.009 (−0.017 to −0.002)	**0.011**
Inferotemporal × time	0.003 (0.0003 to 0.005)	**0.025**	0.005 (0.002 to 0.008)	**<0.001**
Inferonasal	−0.0003 (−0.006 to 0.005)	0.916	−0.0035 (−0.011 to 0.004)	0.363
Inferonasal × time	0.003 (−0.0001 to 0.005)	0.058	0.002 (−0.001 to 0.006)	0.201
Nasal	0.003 (−0.005 to 0.012)	0.411	0.004 (−0.007 to 0.016)	0.440
Nasal × time	0.005 (0.001 to 0.009)	**0.014**	0.007 (0.001 to 0.012)	**0.014**
Superonasal	−0.004 (−0.010 to 0.002)	0.177	−0.004 (−0.012 to 0.004)	0.340
Superonasal × time	0.002 (−0.001 to 0.005)	0.140	0.003 (−0.001 to 0.006)	0.134

*Model A adjusted for: time, age, interaction age/time, sex, school level, Apolipoprotein ε4, diabetes, diameter of optic nerve head, cataract surgery, and glaucoma*.

*Model B adjusted for: the same A-model with additional adjustment for axial length (AL) measure*.

*^a^Additional adjustment for the interaction Apolipoprotein ε4/time in model A and B*.

*MMSE, Mini–Mental State Examination; FCSRT, Free and Cued Selective Reminding Test; RNFL, retinal nerve fiber layer*.

*Bold p-value indicates p < 0.05*.

For *delayed free plus cued recalls*, the decline over the follow-up was also associated with RNFL thickness in the “temporal side”; this association was significant only for the inferotemporal segment in model A (β = 0.003; 95% CI, 0.0003 to 0.005; *p* = 0.025). It was also significant for the three temporal segments in the subset with axial length measured (Model B; β = 0.009; 95% CI, 0.002 to 0.016; *p* = 0.010; β = 0.005; 95% CI, 0.002 to 0.008; *p* < 0.001; and β = 0.005; 95% CI; 0.001 to 0.009; *p* = 0.010, for temporal, inferotemporal and superotemporal segments, respectively). Additionally, thicker RNFL of the nasal segment was also associated with lower decline of the cognitive score (Models A and B). Surprisingly, in model B, an association was also demonstrated between inferotemporal segment thickness and a lower initial score (β = −0.009; 95% CI, −0.017 to −0.002; *p* = 0.011).

Results were globally unchanged in sensitivity analyses excluding participants with glaucoma.

Similar results were also observed in complementary analyses. Participants classified as borderline (12.6%) or outside normal limits (17.8%) for their global RNFL value (i.e., with thin RNFL) according to the Spectralis software classification had a higher decline for the two FCSRT scores of delayed recall (β = −0.40; 95% CI, −0.71 to −0.10; *p* = 0.009 for *delayed free recall* and β = −0.20; 95% CI, −0.34 to −0.05; *p* = 0.007 for *delayed free plus cued recalls*).

## Discussion

We found that, whereas global RNFL thickness was not associated with initial cognitive scores, a thicker RNFL was associated with a lower decline of cognitive performances based on the delayed recall scores of the FCSRT. None of the other cognitive functions were associated with RNFL thickness. These findings suggest that reduced RNFL thickness may reflect a higher risk of episodic memory deterioration over time.

The reduction of RNFL thickness in patients with MCI and/or AD, compared to controls, has been previously reported in several studies (4, 9, 10). These previous studies support a systematic and cumulative reduction of the RNFL thickness with the progression of the cognitive impairment. The association between RNFL and cognition at an earlier stage is largely unknown; yet, cognitive decline is a very long process (25). Thus, our results may fit into a very early stage of cognitive impairment. Moreover, our study takes place within a general elderly population, using a longitudinal design, thus adding complementary knowledge compare to previous studies, which are mostly cross-sectional in clinical settings.

The association shown in our results was specific to episodic memory, which is a cognitive domain strongly implicated in AD process. A previous study evaluating the relationship between RNFL and cognition also found an association between RNFL and episodic memory (word list learning and story recall) among participants with normal cognition (26). However, this was a cross-sectional study with a small sample size (*n* = 52).

No association was found with MMSE score, which reflects global cognition. However, our population is mainly a well-functioning population without dementia at baseline; the MMSE test may be not sensitive enough to detect subtle changes in the early phase of cognitive deterioration (27). Similarly, no correlations between MMSE and peripapillary thickness were found in most previous studies comparing peripapillary RNFL in AD patients and healthy controls (28, 29). Only 1 previous study comparing 35 AD, 35 MCI, and 35 healthy controls reported a significant correlation between peripapillary RNFL and MMSE in an analysis combining all the participants (30).

More surprisingly, we also found no association with Isaacs’ verbal fluency test, even though this test has detected changes during very early cognitive decline, as many as 15 years before the clinical diagnosis of Alzheimer’s dementia (25). However, among elderly people, dementia is the result of several processes including neurodegeneration and vascular damage (31). Cognitive impairment due to vascular processes is believed to preferentially manifest through impaired attention and executive function rather than memory function (32). Therefore, verbal fluency could reflect the global vascular process more than the neurodegenerative process. This may explain the lack of association with RNFL thickness, which may be more strongly related to neurodegeneration.

Our SD-OCT device automatically calculates RNFL thickness both globally and in each of the six segments. This offers the advantage of providing data for the superotemporal and inferotemporal segments, which are the thickest zones in the peripapillary area and the most sensitive zones to early age-related defects (20, 33). As previously reported, the highest RNFL values in our data were found in the inferotemporal and superotemporal segments, which, together with the temporal segment, are also associated with cognitive decline (20).

Our results showing cognitive test alterations reflecting episodic memory changes are consistent with previous reports stating that the primary degenerative process leading to MCI and/or AD is marked by an early deterioration of episodic memory (27, 34, 35). In normal elderly subjects, the evaluation of this deterioration can even predict the time of progression to MCI as well as the time of progression from MCI to AD (36). Furthermore, even though “recall measures” are cited more often as good cognitive markers of future AD, “delayed recall measures” are excellent predictors of progression to AD (37, 38) and can even increase the sensitivity of recall scores used in AD identification (39). Therefore, our findings may be relevant to the specific brain changes found in the hippocampus and temporal regions of people in the preclinical stage of AD (40–42). Previous imaging data showing a correlation between RNFL and cerebral volumes in the temporal and occipital lobes are concordant with these findings (43). In addition to RNFL, future studies should explore the ganglion cell-body layer in relationship with cognitive performance and brain alteration.

Glaucoma was taken into account in our analyses by adjusting for this pathology and by performing sensitivity analyses excluding participants with glaucoma. Glaucoma is a degenerative disease of the optic nerve, and we previously found an increased risk of dementia among participants with glaucoma (11). The inferotemporal and superotemporal segments are the first segments damaged in glaucoma. These segments (and the temporal segment) are also the ones associated with cognitive decline. Therefore, similar neurodegenerative processes could lead to both glaucoma and cognitive decline. Axial length was also adjusted for in our analyses. However, supplementary analyses comparing results with and without adjustment for axial length in the subsample of 204 participants with available axial length data showed that this adjustment did not materially change our results (data not shown); thus, even if axial length is associated with RNFL, it seems not confound the relation between RNFL and cognition.

Our study has several limitations. First, our follow-up period of 2 years may be too short to demonstrate larger effects on other cognitive functions. However, to our knowledge, this is the first longitudinal study with a large cohort in this field. Second, our study included well-functioning subjects who consented to eye examination and who therefore may have a lower decline of cognitive performance over time than the general population. Thus, despite our large study population compared to previous case-control studies in this field, we cannot exclude a possible lack of power. Third, as race was not available in our cohort, we could not adjust for in the analyses; however, our population was mostly from a Caucasian origin. Fourth, data on refractive error, another ocular variable known to influence RNFL (44) was not included in our analyses; but given our findings on axial length it is unlikely to have affected results. Finally, as RNFL measurement was not available at the baseline time of the Alienor study, the effect of the RNFL evolution over time on cognition could not be studied; further follow-ups of our cohort will allow studying this.

Our study has some strengths. It is based on a longitudinal design with a large population-based cohort including nearly 430 elderly participants. It includes a systematic eye examination that allows the identification and the exclusion of ocular pathologies likely to influence RNFL measurement (vitreomacular traction, myelinated retinal nerve fibers, peripapillary choroidal neovascularization, or myopic chorioretinopathy). The OCT assessments were performed on the same OCT machine by the same experienced technician following a standardized protocol. Moreover, the spectral domain technique provided the best quality and reproducibility currently available (45). Cognitive functions were systematically assessed by trained psychologists blinded to ophthalmologic data using standardized procedures and evaluating several cognitive domains. The association between RNFL and delayed episodic memory was found with both RNFL as a continuous variable and using the threshold provided by the device to classify participants as borderline or outside normal limits for RNFL. Finally, numerous potential confounding factors were taken into account.

## Conclusion

Our findings enrich the literature on the potential utility of RNFL in cognitive deterioration. Although there are still gaps in detailed knowledge of the early association between neurodegeneration in the retina and in the brain, our findings could lead to the next steps of knowledge in that domain. However, these results should be corroborated with larger populations and longer follow-ups. Moreover, cerebral imaging studies should be implemented to better understand the association between retinal and brain neurodegeneration.

## Author Contributions

JM-G analyzed the data and draft the first version of the manuscript. CH, CD, J-FD, J-FK, and JM-G designed the study. M-BR, J-FK, CS, M-ND, HA, J-FD, CD, and CH acquired the data. All the co-authors contributed to the interpretation of the results, critically revised the manuscript, and approved the final version.

## Conflict of Interest Statement

JM-G, LT, and HA disclose no conflict of interest. M-BR is consultant for Allergan, Bausch & Lomb. She has received funding for conferences from Biogen, Thea, Novartis. CS has received funding for travel from Alcon, Allergan, AMO. M-ND has received funding from laboratoires Théa (advisory board, travel funding) and Novartis (advisory board, travel funding). J-FK is consultant for Alcon, Allergan, Bayer, Carl Zeiss Meditec, Novartis, Théa. J-FD serves on a scientific advisory board for and has received funding for travel from Janssen-Cilag; received a gift worth more than US$500 from Novartis; holds a corporate appointment with Merck Serono; and has received research support from Novartis and Ipsen. CD has received funding from laboratoires Théa (grant, advisory board, travel funding), Novartis (consultant), and Bausch & Lomb (consultant). CH has received honoraries from Novartis.
