# Supplementary material for: Peripapillary Retinal Nerve Fiber Layer Thickness and the Evolution of Cognitive Performance in an Elderly Population
Source: Front Neurol. 2017 Mar 21;8:93. doi: 10.3389/fneur.2017.00093 (PMC5358525; doi:10.3389/fneur.2017.00093)
Supplement: Supplementary file 1 [file Data_Sheet_2.pdf]

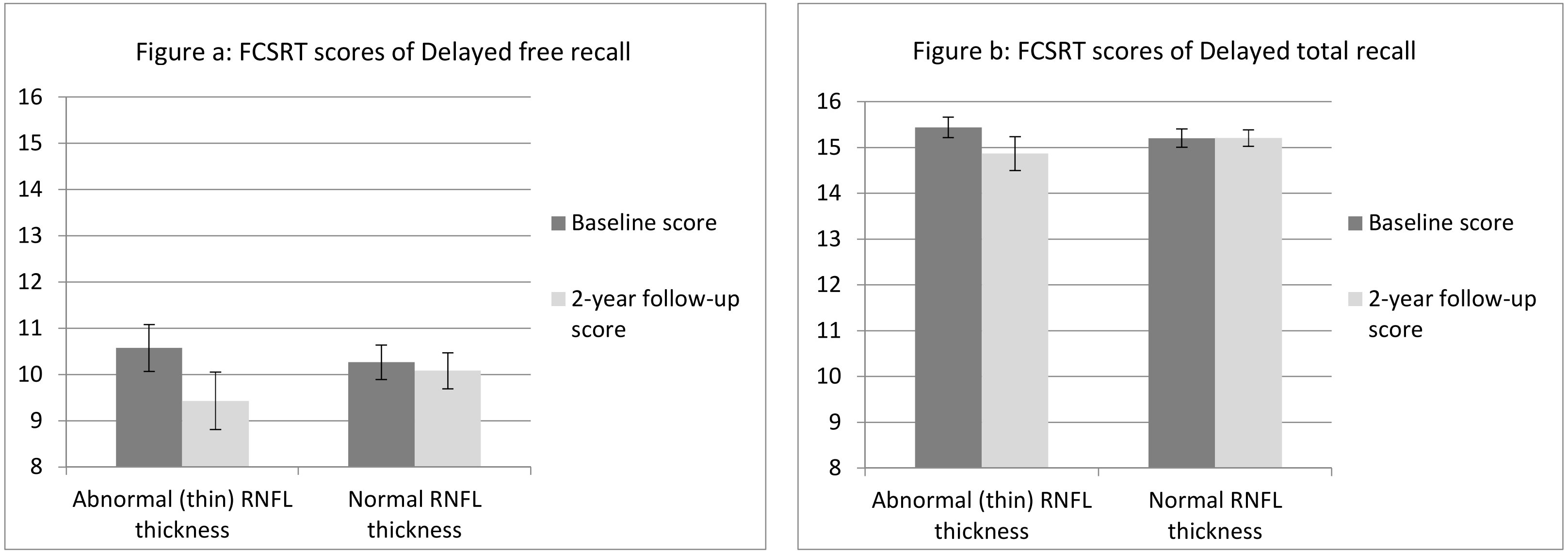

Supplementary Figure: Mean (and 95%CI) FCSRT score at baseline and at the 2-year follow-up according to RNFL value at baseline. Figure a: Delayed free recall score. Figure b: Delayed total recall score

FCSRT: *Free and Cued Selective Reminding Test*

95%CI: *Confidence Interval at 95%*

RNFL*: Retinal Nerve Fiber Layer*

Abnormal and normal RNFL thicknesses are provided by the Spectralis software: abnormal values (including outside normal limits (lowest 1% of the distribution) and borderline (bottom 1% to 5% of the distribution)); and within normal (above 5%) based on the age-matched comparison with an internal normative database of 201 healthy eyes of Caucasian patients. For each participant, the worse eye is considered here.

Supplementary table: Mean decline of cognitive performance between baseline and follow-up assessment, N=427.

Cognitive assessment tests	Mean difference score (follow-up score - baseline score)	95% CI of the mean difference	*p**
MMSE, mean (SD)	-0.22	-0.40; -0.04	*0.015*
Isaacs' set test at 30", mean (SD)	-2.09	-2.72; -1.46	<*.0001*
FCSRT, mean (SD)			
Score of *three free recalls*	-1.19	-1.78; -0.61	<*.0001*
Score of *three free plus cued recalls*	-1.02	-1.39; -0.65	<.0001
Score of *delayed free recall*	-0.55	-0.80; -0.30	<.0001
Score of *delayed free plus cued recalls*	-0.25	-0.40; -0.10	0.0009

* Student t-test for paired data

SD*: Standard deviation*
